# PREDICTORS OF EVERYDAY FUNCTIONING, ADLS/IADLS AMONG OLDER ADULTS USING THE ACTIVE STUDY

**DOI:** 10.1093/geroni/igad104.3112

**Published:** 2023-12-21

**Authors:** Deborah Dadson, Karlene Ball

**Affiliations:** University of Alabama, Birmingham, Birmingham, Alabama, United States; University of Alabama at Birmingham, Birmingham, Alabama, United States

## Abstract

The increase in life expectancy over the years has triggered both researchers and the aging population to focus more on achieving an active engagement with life. This ability to perform daily living activities (functionality) is a key component in healthy aging which aids older adults live independently with or without disease and disability. Gaining insight into the key elements that enhance physical functioning is necessary as the issue of limited physical functioning affects not only older adults but also, middle aged adults. This study examines the predictors of everyday functioning, Activities of Daily Living (ADLs) and Instrumental Activities of Daily Living (IADLs) among 1920 older adults using data from the Advanced Cognitive Training for Independent and Vital Elderly (ACTIVE) study. ADLs and IADLs were self-reported. Results of the hierarchical linear regression at baseline indicate that, being female and being African American significantly predict lower performance on everyday functioning, after adjusting for age and years of education. Fewer falls in the past 2 months, better cognition and normal weight significantly predict high performance on everyday functioning (all p < .05). For ADLs, being African American and female gender significantly predict higher scores on ADLs while female gender predicted lower scores on IADLs. There were no differences on living arrangements and access to healthcare on everyday functioning, ADLs and IADLs. 43.1% of variability in IADLs were explained by the predictors. Findings from this study identify target populations for interventions to promote active engagement with life.

